# Public Perception towards Waste-to-Energy as a Waste Management Strategy: A Case from Shandong, China

**DOI:** 10.3390/ijerph16162997

**Published:** 2019-08-20

**Authors:** Xueliang Yuan, Xiaohan Fan, Jiaxin Liang, Mengyue Liu, Yuqiang Teng, Qiao Ma, Qingsong Wang, Ruimin Mu, Jian Zuo

**Affiliations:** 1 School of Energy and Power Engineering, Shandong University, Jinan 250061, China; 2 New Oriental Education and Training School, Xiamen 361021, China; 3 School of Municipal and Environmental Engineering, Shandong Jianzhu University, Jinan 250100, China; 4 School of Architecture & Built Environment, The University of Adelaide, Adelaide 5005, Australia

**Keywords:** sustainable development, social acceptance, municipal solid waste, incineration

## Abstract

Municipal solid waste (MSW) is posing great challenge for most countries in the world, which can cause severe negative impacts to the environment and human health. Waste-to-energy has great potential in China because of its technological maturity and policy support at the national level. However, there are significant conflicts between the huge market demand and strong public opposition. It is imperative to examine the public perception of waste-to-energy, especially for developing countries where a large number of projects are under construction or have been approved. The public perception of waste-to-energy was carried out by a questionnaire survey in this research. A total of 650 questionnaires were distributed and 629 questionnaires were returned, with a response rate of 96.8%. The results show that the public showed general concern in regard to environmental issues. Respondents had an overall positive attitude towards waste-to-energy, but it varied according to the demographic details of residents, such as age, education, and income. Recognition level of the benefits was higher than the concern of associated risks. Multiple linear regression shows that awareness of environmental issues had no impact on public attitude towards waste-to-energy, while public awareness and perceived benefits had notable positive impacts. Perceived risks had a positive correlation with public attitude. In order to promote the development of MSW incinerators, the government should make more publicity efforts. Rural residents, people over 50 years old, and people with low education and low income are the major groups which should be focused on to enhance the public perception. The findings provide a theoretical and practical reference for enhancing the social acceptance of waste-to-energy development.

## 1. Introduction

With the rapid growth of the urban economy and population in China, the output of municipal solid waste (MSW) has dramatically increased, becoming a constant threat to residents’ living environments and health [1]. The negative impacts of MSW include land occupation, environmental pollution, and spread of disease. Waste-to-energy (WTE) has the advantages of good volume-reduction effect, small land occupation, stability, and minor secondary pollution [2]. It can meet environmental emission standards under good operation and strict process control. In addition, it is a new source of energy generation [3]. As one of the most effective means of disposal currently and in the near future, WTE has social, environmental, and economic benefits [4]. Meanwhile, national policies highly support the development of WTE projects in China.

However, there is lack of public awareness and understanding of WTE, unfortunately leading to not-in-my-back-yard (NIMBY) syndrome [5]. A WTE project in Guangzhou was called off due to serious protests by local residents, even though the project passed the official environmental impact assessment [6]. Some similar protests occurred in the Zhejiang, Hubei, Hunan, and Hainan provinces in China in recent years [7]. Although the adoption of the waste-burning policy was not much debated in Taiwan, the sitting of incinerators has been vigorously opposed by community-based protest movements [8]. This indicated the lack of public awareness and the misunderstanding of WTE. It also shows that public attitude is crucial to the implementation of WTE projects. However, another WTE project in Zhejiang province is also being regarded as the first one that truly overcame the NIMBY issue in China, which reveals that a publicly involved approach played an important role in the success of the decision-making [9].

WTE is a source of energy, and is a new technology compared with other methods for fossil energy utilization. Renewable versus non-renewable energy sources and their respective environmental impacts have emerged as preeminent industrial and environmental concerns [10]. The social acceptance of this sector has drawn wide attention in recent years. Social acceptance presents a constraint on the increase of new and renewable energy share in many countries [11]. Yuan et al. examined the social acceptance of solar energy technologies from the end user’s perspective in China [12]. Their survey identified the main uses and purposes of the solar water heater and solar photovoltaic, highlighting the key sections that can be improved. The public acceptance of marine renewable energy (MRE) in Malaysia revealed a positive overall attitude to MRE, but with NIMBY [13]. The NIMBY phenomenon also exists for carbon capture and storage technology in Holland [14]. A survey of the social acceptance of nuclear power in China revealed a higher degree of environmental concern, and indicated the challenge of nuclear power developers [15].

Some studies also investigated MSW-related issues from different perspectives. The most sustainable cities in EU considered that energy recovery from wastes was a critical factor for their sustainability [16]. In Zimbabwe, waste management still remains a key social and engineering concern due to the health hazards associated with poor waste treatment [17]. In Malaysia, significant efforts have been made to utilize MSW for energy production by employing a gasification process [18]. A comprehensive survey of the energy recovery potential from MSW in Brazil shows energy recovery is among the actions foreseen for environmentally appropriate disposition of solid waste [19]. Bhuvaneswari and Sudha identified the awareness about the effects of improper household waste management and governmental measures [20]. They argued that there is an urgent need to understand the importance of waste management. Meanwhile, in response to the carbon emissions from waste incineration, Kang et al. is developing a Korean emissions inventory to estimate greenhouse gas emissions from waste incineration [21]. A questionnaire survey aimed to investigate local residents’ concerns about solid waste management (SWM) facilities, and assessment of the attitudes toward such facilities was carried out in Japan. Obvious correlations were not found between individual items of concern and attitudes to the construction of such a facility [22]. As per a study conducted by Udawatta, both human factors and technical factors act as barriers to implementing SWM practices in Australian construction projects [23]. As a good example for waste management, Switzerland’s success lies in the active reduction and classification of garbage by residents, which has high requirements for residents’ environmental awareness [24]. In China, as per a study conducted by Han, broad and sustainable public willingness to pay and willingness to participate are the basis of successful MSW management [25]. Meanwhile, the attitude and perceived behavior control also influences the willingness for household waste management [26].

As per a study conducted by Jones, it is necessary to explore the influence of social factors prior to policy implementation of SWM [27]. A theoretical psychosocial model was proposed to guide this process in Portugal, which highlighted the joint effect of mediating variables (e.g., environmental annoyance and risk perception) and moderating variables (e.g., attitudes towards the incinerator and local identity) [28]. Enhancing sustainability in solid waste management requires options that alleviate environmental issues and provide economic and social benefits [29]. Lima examined the predictors for the acceptance of waste incineration facilities via a questionnaire survey with a random sample of the residents. The residents’ attitude towards the incinerator was partially mediated by perceived risk, perceived justice, and expectations [30]. A questionnaire survey for the development of a WTE plant in Greece revealed that public attitude on the integration of MSW thermal treatment in the local waste management strategy was positive, but the NIMBY syndrome was obvious [31]. As per a study conducted by Ryba which examined the influence of a planned WTE project on residents’ attitude and the strength of the NIMBY effect, the offered compensation did not have a significant influence on residents’ attitudes [32]. Another piece of research showed that for every additional kilometer the property is away from a WTE plant, the real estate value can increase by 1.30% [33]. Solid waste mismanagement is a social burden that requires the introduction of reliable public policies and technological facilities [34]. The role of citizen participation must be addressed to gain public support for incinerator options in managing solid waste. An effective garbage collection service network design can help to reduce the municipal operation cost and improve its service level [35]. Meanwhile, the NIMBY and social conflict of waste treatment plants are characterized by the interweaving of natural factors and social factors, and should be solved by the combination of environmental science and the social conflict theory [36], as well as good governance [37].

The critical factors to the public acceptance of waste policies by local governments operating incineration facilities were highlighted. The public concerns were well beyond the high risks from pollutants that are discharged from incineration facility. Rather, residents are more anxious about the credibility of the administration at work. A comparative case study of two waste incineration projects, that is, where one succeeded and one failed, found that environmental concerns were the dominant cause for local oppositions, while 70% of respondents supported waste incinerators in general [38]. The Indian government has attempted to curtail this problem by promoting sustainable management methods and strengthening government interventions and policies [39]. A study conducted by Ren assessed the public acceptance of a WTE facility among local residents through a structured questionnaire survey [40]. They characterized strong protesters by socioeconomic status and geographical distribution, and differentiated the socioeconomic status of protesters and supporters of compensation. The effectiveness of any MSW management scheme and its smooth operation heavily depends on its acceptance by the local community. Increasing the participation, raising the knowledge of individuals, providing appropriate facilities and equipment, and implementing coordinated coherent programs of recycling by the governmental and private sectors played a very important role in solid waste management [41,42].

It is contradictory that the MSW incinerator developments receive vigorous promotion from the government and the strong opposition of the residents. It is urgent and necessary to investigate the social acceptance issues which have been recognized as a critical factor for the successful development and operation of WTE projects. This is particularly the case in developing countries such as China, where a large number of WTE projects are under construction or have been approved [43]. Some studies have attempted to address social acceptance issues in MSW incinerator developments. However, the vast majority of these studies did not consider the potential impacts of the demographic profile of the respondents, such as age, gender, education, income, distance from the facility, and the source of respondents. Furthermore, few studies focused on the integrated analysis for the general environmental issues, public awareness (PAW), public attitude (PAT), as well as the perceived risks (PR) and perceived benefits (PB) of WTE. This research aims to address these gaps by a case study in China. The findings will be useful for evidence-based policy-making in future MSW incinerator developments.

## 2. MSW in China and the Policy System

MSW has become a serious problem in China over the course of the last two decades, resulting in significant side-effects to the environment [44]. In China, 201.9 million tons of MSW were generated in 202 cities in 2017 [45]. It is estimated that the amount of urban domestic waste generated in China will reach 222.2 million tons in 2019. With rapid urbanization and improvement of living standards, the production of MSW will keep its rapid growth in China [44,46].

**Figure 1:**
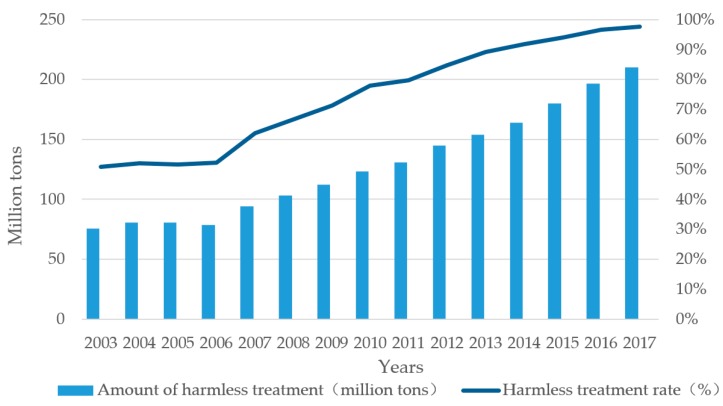
Harmless treatment of municipal solid waste (MSW) in China in the past 15 years.

The MSW collection and transportation system has become more effective in the past 15 years. The harmless treatment of MSW, which includes three major methods—that is, incineration, sanitary landfill, and composting, has been significantly improved (see Figure 1) [47]. WTE capacity will increase from 235.2 thousand t/d in 2015 to 591.4 thousand t/d in 2020.

**Figure 2:**
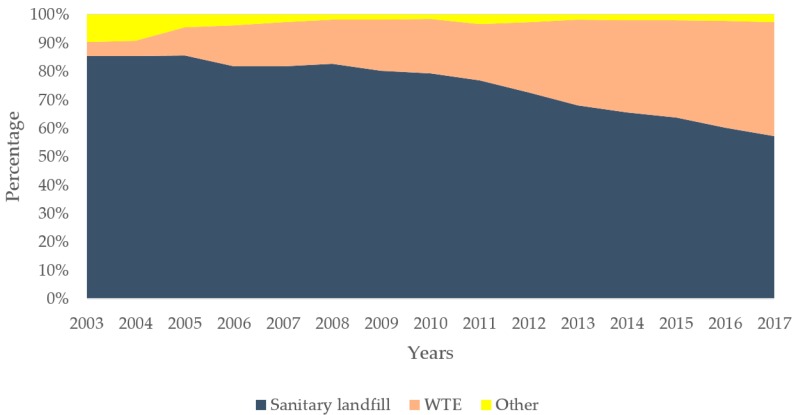
The proportion of MSW treatment methods in China in the past 15 years.

**Figure 3:**
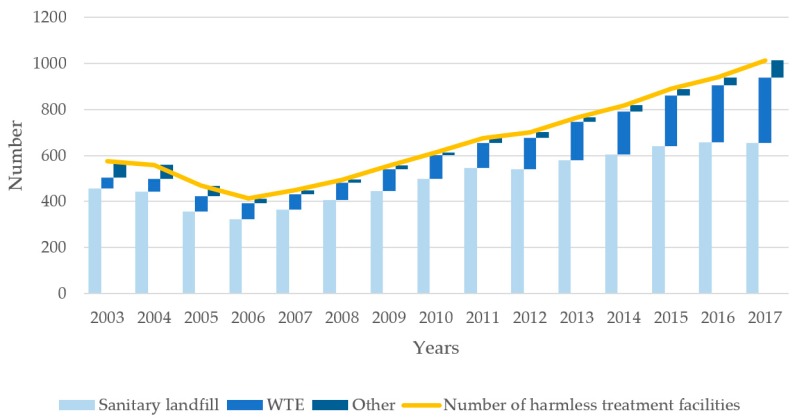
Number of harmless treatment facilities of MSW in China in the past 15 years.

The methods for MSW treatment and the corresponding treatment facilities in China have changed significantly (see Figure 2 and Figure 3). The percentage of sanitary landfill decreased from 84.88% in 2003 to 57.23% in 2017. The number of facilities for sanitary landfill simultaneously show a slow increase from 457 to 654—however, the percentage of WTE increased from 4.90% to 40.24% in the past 15 years. The number of incineration plants show a rapid growth from 47 to 286 in the same period [48]. Compared with the developed countries, MSW treatment in China is still at the initial stage, mainly based on sanitary landfill. At present, about two-thirds of the cities in China have problems with waste disposal [49]. One-fourth of the cities do not have suitable landfill sites, and MSW have accumulated more than 5 × 10^8^ m^2^ of land. Waste incineration is one of the most effective ways to deal with domestic waste in China. By the end of 2020, WTE capacity will account for more than 50% of the total capacity of harmless treatment, of which the eastern region will reach more than 60% and even achieve “zero landfill” in some cities [47].

In terms of the policy system, China has promulgated 35 laws and regulations directly related to MSW disposal, such as the Law of Solid Waste Environmental Pollution Control, and the Regulation on the Administration of City Appearance and Environmental Sanitation. A total of 68 standards related to MSW have been released. These include five national standards and 63 industrial standards, such as the Pollution Control Standard for WTE, and Technical Specification for WTE Treatment Engineering. The potential environmental pollution of WTE is one of the main public concerns. The Chinese government issued four specific environmental standards related to the air pollutant emissions of WTE (see Table 1). The concentration limits are getting more and more stringent with the continuous technological innovation of WTE and pollution control [50]. For instance, compared with HJT18-1996, the maximal emission concentration of particulate matter (PM), NO_X_, SO_2_, and HCl in GB 18485-2014 decreased by 70%, 40%, 66.7%, and 88%, respectively. The emission concentration of Hg and its compounds, Pd and others, and PCDDs also decreased by 75%, 37.5%, and 90% in the latest environmental standard. Most environmental indicators for WTE in China are now in line with the international requirements.

**Figure 4:**
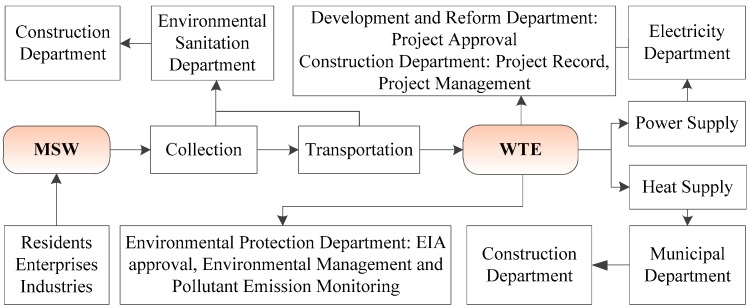
The procedure of a waste-to-energy (WTE) facility and the corresponding government authorities.

At the national management level, the Chinese government attaches great importance to the standardized management of WTE development. Figure 4 shows the procedure and the corresponding government authorities for MSW collection, transportation, and incineration. The construction authority is responsible for the general management of MSW disposal facilities. The environmental protection authority is responsible for the approval of environmental impact assessment, environmental monitoring, and management of the facilities.

## 3. Methods

In order to investigate the social acceptance issues associated with WTE, a questionnaire survey was undertaken. The first section in the questionnaire was designed to collect the demographic information of respondents, such as gender, age, education, annual income, and distance from the WTE project to the residency (Table 2). The second section was designed to examine various aspects of social acceptance. There are 29 questions in five groups: (1) PAW of the environmental issues (Q1–Q2), (2) PAW towards WTE (Q3–Q6), (3) PR of WTE (Q7–Q15), (4) PB of WTE (Q16–Q24), and (5) PAT towards WTE (Q25–Q29). The draft questionnaire was pilot-tested with 30 local respondents in Jinan City. The feedback was overwhelmingly positive, and no change was made. The research team randomly selected the respondents from the houses, apartments, and main streets in urban and rural areas of Jinan City and Liaocheng City in Shandong Province. Jinan is the capital city and one of the economically developed cities of Shandong Province. Liaocheng is one of the economically underdeveloped cities in western Shandong. These two cities are representative in the aspect of different economic development levels. In the process of the questionnaire survey, some respondents were tourists, rather than local residents. The tourist group was also included in this research. This enriched the contents of cross-group comparisons. A total of 650 questionnaires were distributed, and 629 questionnaires were returned (see Table 2).

The reliability, correlation, and missing values of questionnaire data were analyzed by SPSS 17.0. Sample data was determined by a five-point Likert method—that is, 1 = Strongly agree, 2 = Agree, 3 = Neutral, 4 = Disagree, and 5 = Strongly disagree. In order to determine the significance of different groups and discuss the difference according to the mean value, data were analyzed by analysis of variance (ANOVA), student’s *t* test (*t*-test), and multiple regressions. ANOVA was used for the significance test of the mean difference between samples. The *t*-test used the *t*-distribution theory to deduce the probability of differences to compare whether the difference between two mean values is significant or not. Multiple regression was used to describe the correlation between one dependent variable and multiple independent variables.

## 4. Results and Discussions

### 4.1. PAW of Environment Issues

As shown in Table 3, respondents were widely concerned about environmental issues. The mean value of “environmental pollution issues” is slightly lower than “the shortage of resources and energy”. This indicates that the PAW level of environmental pollution issues is higher than that of the shortage of resources and energy, but the differences are not significant. The percentage of respondents holding the views of “Disagree” and “Strongly disagree” is very low.

### 4.2. PAW Towards WTE

The mean values shown in Table 4 indicate that the awareness of “WTE technology” and “the national policies about WTE” is relatively low. Q3–Q6 were the dependent variables used to perform the ANOVA and t-test. The significance level was set as 0.05. The results are listed in Table 5.

**Figure 5:**
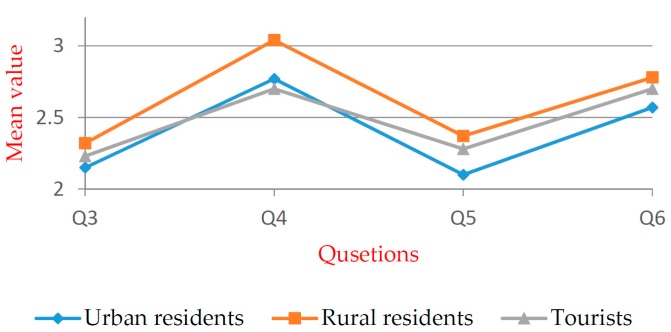
Mean values of “respondent source” group.

**Figure 6:**
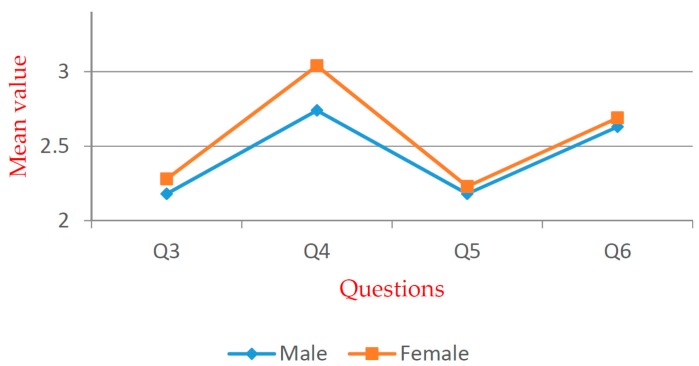
Mean values of “gender” group.

**Figure 7:**
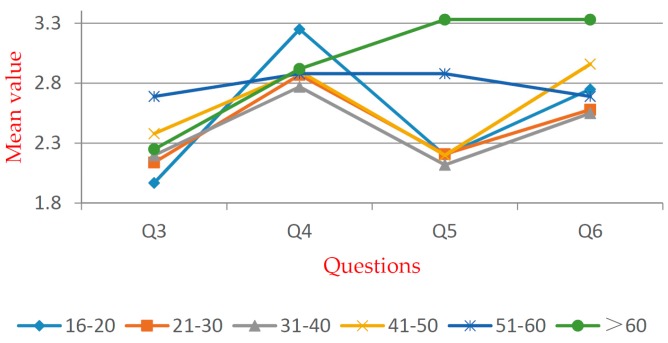
Mean values of “age” group.

**Figure 8:**
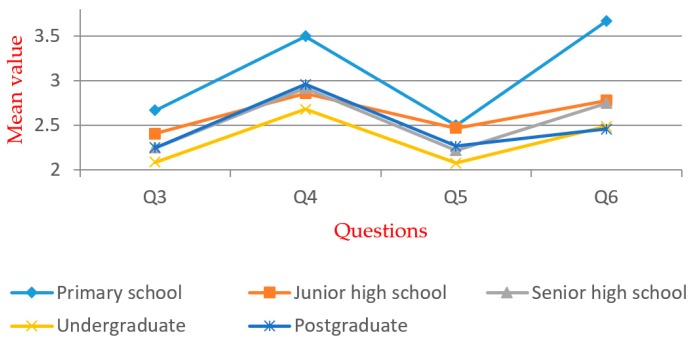
Mean values of “education” group.

**Figure 9:**
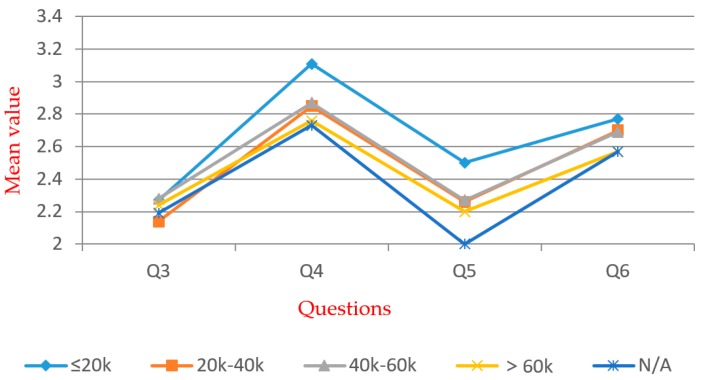
Mean values of “income” group.

**Figure 10:**
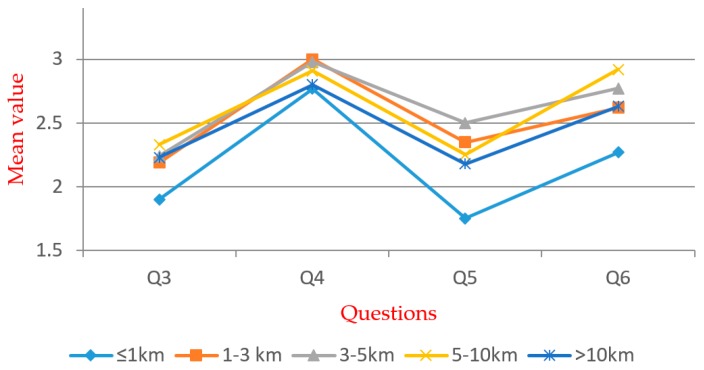
Mean values of “distance” group.

Table 5 shows that significant differences exist within all groups. For Q5, significant differences exist within the groups except for the “gender”. Multiple comparison and independent exponent-tests were performed. The mean values are shown in Figure 5, Figure 6, Figure 7, Figure 8, Figure 9 and Figure 10.

Figure 5 shows significant differences in all groups. There are significant differences between urban residents and rural residents in Q3–Q6, but the urban residents’ mean value is less than the rural residents’ mean value. This indicates that the awareness level of urban residents in regard to WTE technology, the possible risks, and the related policies is higher than those of rural residents. The significant difference between rural residents and tourists in Q4 indicates a higher level of awareness of WTE technology from tourists.

According to the t-test, the awareness and attitude of urban residents is more positive than those of rural residents. Efforts are required to educate rural residents about the knowledge of WTE. The *t*-test results show a significant difference in Q4, and the mean value of males is less than that of females (see Figure 6). This indicates that male residents have a better knowledge of WTE technology than females.

In the “age” group (see Figure 7), significant differences exist within Q3, Q5, and Q6 by one-way ANOVA. According to the *t*-test, the over-60s group reported the largest mean value in Q5 and Q6; the “50–60” group reported the largest mean value in Q3. It indicates that older respondents have a lower level of awareness and acceptance to WTE. In the “education” group (see Figure 8), significant differences exist within Q3–Q6 by one-way ANOVA. According to the *t*-test, the awareness level of well-educated groups is generally higher than that of the less-educated groups. It shows that education is positively related to the degree of social acceptance. Therefore, the government and related stakeholders should put more emphasis on less-educated people in order to promote WTE projects.

In the “annual income” group (see Figure 9), significant differences exist among Q4 and Q5, as indicated by one-way ANOVA. According to the *t*-test, the “≤20k” group has lower acceptance. The “N/A” group is the opposite, with a relatively high awareness level. Other income groups do not have a significant difference in Q3–Q6, which shows that income has less of a positive correlation with awareness toward WTE, except the group with the lowest income. Thus, in order to improve the overall public awareness for WTE, focus should be placed on the “≤20k” group.

In the “distance” group (see Figure 10), significant differences exist within Q3, Q5, and Q6. According to the *t*-test, the “≤1 km” group has a much higher acceptance rate than other groups. Other groups do not have a significant difference, except for the “3–5 km” group, with relatively lower acceptance. As a result, the “≤1 km” is the most positive group in awareness towards WTE. This group has much more opportunities to learn the information or get hired by the WTE plants. The supporting infrastructure of WTE plants also provide convenience to this group.

### 4.3. PR Associated with WTE

Despite a number of benefits, there are various issues associated with WTE, such as high investment, waste gas, waste liquid, waste residue, noise, and odor [51,52,53]. Table 6 shows that how “WTE may produce obvious odor pollutants” is one of the risks that respondents mostly worry about, followed by the risk that “WTE will impact the health of residents”. Respondents were least concerned about “noise pollution”. Odor control must be strengthened in the processes of WTE.

The WTE plant is one of the major targets that was strictly supervised and inspected by the environmental protection authority to make sure the pollutants emissions met the national and local environmental standards. All WTE plants are equipped with an online monitor system. The pollutants emission realized real-time monitoring. However, the waste collection and transportation cannot be strictly monitored in the whole process. Leachate leakage and odor pollutants may occur in the transportation and the storage stage. This echoes the result of the questionnaire survey.

### 4.4. PB of WTE

The advantages of WTE are recognized from the perspective of ecological protection and energy generation [54,55]. In addition, the development of WTE plants will also provide employment opportunities. Comparing the data in Table 6 and Table 7, all of the mean values of the PB are lower than PR. This indicates that the respondents are generally concerned about the risks associated with WTE, despite recognizing benefits. “WTE can solve garbage siege” was ranked as the most significant benefits of WTE, followed by “provides jobs” and “social benefits”. In general, respondents considered WTE as an effective method for solid waste disposal and ecological protection.

### 4.5. PAT Towards WTE

As shown in Table 8, survey respondents preferred electricity by WTE rather than coal-fired power. However, they showed preference of “construction in nonlocal place” than “construction in local place”.

The respondents were classified according to their source, gender, age, education, income, and distance to the MSW incineration project. Sample data were analyzed by multiple linear regression analysis in SPSS with the following equation. The significance level was set as 0.05. Results are listed in Table 9.

*PAT = F1 (source, gender, age, education, income, distance)* (1)

As shown in Table 9, “age” and “distance” show a positive correlation to PAT, while “education” and “income” show a negative correlation. With the increase of age and distance, PAT becomes negative. With the increase of education and income, PAT becomes positive.

### 4.6. Impact of Other Issues on PAT

Sample data were analyzed by using multiple linear regression analysis. PAT was taken as a dependent variable, while the PAW of environmental issues—PAW, PR, and PB—were taken as independent variables. The significance level was set as 0.05. Results are shown in Table 10. The following is the multiple regression equation.

*PAT = F2 (PAW of environmental issues, PAW, PR, PB)* (2)

As shown in Table 10, the PAW of environmental issues is not significant for PAT. PAW and PB show a positive correlation to PAT. This means a higher PAW and PB level will result in a higher acceptance level. As for PR, the positive correlation with PAT is negligible.

## 5. Conclusions

WTE is developing rapidly in China. However, there are significant conflicts between the support of the government and the objection of local residents. Social acceptance has become a crucial factor for the development of WTE. This research shows that the public have a high level of awareness for environmental and resource issues. The respondents understand the benefits of WTE, but know less about WTE technology and the related policies, leading to the concern about the risks of WTE. Odor pollutants and health impacts are two of the most concerning issues. This echoes why respondents show a more positive attitude to “non-local construction” than “local construction”. The NIMBY syndrome is obvious, which is consistent with previous studies.

The one-way ANOVA and *t*-test show that the awareness level of urban residents is higher than that of rural residents. Respondents above 50 years old were found to have a lower awareness in regard to WTE. The group of annual income that was “≤20 k” had a relatively low awareness level, while the difference for other income groups were not significant. The group with a living distance of “≤1 km” had a much higher degree of awareness than other groups. Categorical analysis showed that an increase in “age” and “distance” caused a lower acceptance level, while higher “education” and “income” levels led to a higher acceptance level. Multiple linear regression showed that awareness of environmental issues did not have significant impacts on PAT towards WTE. By contrast, PAW and PB had significant positive impacts on PAT. PR had a negligible positive correlation with PAT.

In order to promote the development of MSW incinerators, the government should make more publicity efforts. The technical knowledge, environmental standards, and regulatory policies should be popularized to the residents. Rural residents, people over 50 years old, and people with low education and low income are the major groups which should be focused on to raise social acceptance. Furthermore, the authorities should further standardize and normalize the collection, transportation, and storage system of MSW, strengthen environmental supervision, and increase the transparency of information.

## Figures and Tables

**Table 1:** Standard pollutants emission limits for different periods in China.

Pollutants	Unit	Small Incinerator Pollutant Emission Standard	Domestic Waste Incineration Pollution Control Standard	Domestic Waste Incineration Pollution Control Standard
		HJT18-1996	GB18485-2001	GB18485-2014 (Effective)
PM/soot	mg/m^3^	100 (mean value)	80 (mean value)	30 (hourly mean)
NOX	mg/m^3^,	500	400	300
	hourly mean			
SO2	mg/m^3^,	300	260	100
	hourly mean			
HCl	mg/m^3^,	500	75	60
	hourly mean			
Hg and its compounds	mg/m^3^,	\	0.2	0.05
	mean value			
Cd + Ti	mg/m^3^,	\	0.1	0.1
	mean value			
Pd and others	mg/m^3^,	\	1.6	1.0
	mean value			
PCDDs	mg/m^3^,	\	1.0	0.1
	mean value			
CO	mg/m^3^,	1000	150	100
	hourly mean			

Note: Domestic waste incineration pollution control standard GWKB 3-2000 is the revision of Small Incinerator Pollutant Emission Standard HJT18-1996, but it was replaced by Domestic waste incineration pollution control standard GB18485-2001 one year later. These two standards have the same contents. The later one is displayed in this table.

**Table 2:** The demographic information of respondents.

Respondent Source	Gender	Age	Education	Annual income	Distance *
Urban Resident (368)	Male (406)	16–20 (40)	Primary school (6)	≤20k RMB (98)	≤1 km (48)
Rural Resident (197)	Female (223)	21–30 (125)	Junior high school (51)	20–40k RMB (117)	1–3 km (37)
Tourist (64)		31–40 (339)	Senior high school (310)	40–60k RMB (134)	3–5 km (62)
		41–50 (98)	Undergraduate (210)	>60k RMB (47)	5–10 km (79)
		51–60 (16)	Postgraduate (52)	N/A (233)	>10 km (403)
		>60 (11)			

Note: Figures in brackets stand for the number of respondents. * The distance between residence of the respondents to WTE facilities.

**Table 3:** The mean value of public awareness (PAW) of the environmental issues.

Number	Question	Mean Value *
Q1	You care about environmental pollution issues	1.76
Q2	You care about the shortage of resources and energy	1.81

* Mean value = (1 * the number of respondents who strongly agree + 2 * the number of respondents who agree + 3 * the number of respondents who choose neutral + 4 * the number of respondents who disagree + 5 * the number of respondents who strongly disagree)/629.

**Table 4:** The mean value of PAW towards WTE.

No.	Question	Mean Value
Q3	You know how the MSW is disposed	2.21
Q4	You know WTE technology	2.84
Q5	You know WTE may cause environmental pollution	2.20
Q6	You know the national policies about WTE	2.65

**Table 5:** The significance level of PAW towards WTE.

No.	Source	Gender	Age	Education	Income	Distance
Q3	0.032 *	0.092	0.005 *	0.014 *	0.527	0.028 *
Q4	0.007 *	0.001 *	0.134	0.042 *	0.045 *	0.539
Q5	0.001 *	0.434	0.000 *	0.019 *	0.000 *	0.000 *
Q6	0.040 *	0.501	0.001 *	0.000 *	0.405	0.003 *

Note: * stands for the significant *p*-value.

**Table 6:** Mean value of the PR of WTE.

No.	Risks	Mean Value	Rank
Q11	WTE may produce obvious odor pollutants	2.40	1
Q12	WTE will impact the health of residents	2.51	2
Q13	The transport process has environmental impacts	2.53	3
Q14	The storage process has environmental impacts	2.59	4
Q15	The ash disposal process has environmental impacts	2.60	5
Q7	WTE may emit air pollutants	2.62	6
Q8	WTE may discharge waste liquid	2.66	7
Q9	WTE may produce solid waste	2.71	8
Q10	WTE may have noise pollution	2.77	9

**Table 7:** The mean value of PB of WTE.

No.	Benefits	Mean Value	Rank
Q24	WTE can solve garbage siege	1.93	1
Q20	WTE can provide jobs	1.96	2
Q16	WTE have significant social benefits	1.98	3
Q18	WTE have significant environmental benefits	2.07	4
Q17	WTE have significant economic benefits	2.07	5
Q21	WTE can increase the income of local residents	2.10	6
Q23	WTE can improve local environmental quality	2.12	7
Q22	WTE can reduce the consumption of fossil fuel	2.14	8
Q19	WTE can promote economic development	2.15	9

**Table 8:** The mean value of PAT towards WTE.

No.	Questions	Mean Value	Rank
Q27	You prefer the electricity from WTE rather than coal	2.17	1
Q28	You support WTE construction in nonlocal place	2.20	2
Q25	You think WTE has a bright prospect	2.27	3
Q29	You support WTE construction in local place	2.30	4
Q26	You think WTE is the best way to dispose waste	2.48	5

**Table 9:** B-values of related independent variables of categorical multiple regression analysis.

No.	Source	Gender	Age	Education	Income	Distance
Q25	/	/	0.136	/	−0.112	0.132
Q26	/	/	0.093	−0.104	−0.054	0.078
Q27	/	/	/	/	−0.089	0.101
Q28	/	/	/	/	−0.081	0.129
Q29	/	/	/	/	−0.064	0.023

Note: “/” stands for non-significant linear regression.

**Table 10:** B-values of independent variables of multiple regression analysis.

Independent Variables	PAT
PAW of environmental issues	/
PAW	0.442
PR	0.055
PB	0.375

Note: “/” stands for non-significant linear regression.

## References

[1] Li X.R., Bi F., Han Z.D., Qin Y., Wang W.X. (2019). Garbage source classification performance, impact factor, and management strategy in rural areas of China: A case study in Hangzhou. Waste Manag..

[2] Udomsri S., Martin A.R., Martin V. (2011). Thermally driven cooling coupled with municipal solid waste-fired power plant: Application of combined heat, cooling and power in tropical urban areas. Appl. Energy.

[3] Hutton B., Horan E., Norrish M. (2013). Landfill, Compost or Incineration? Finding the Best Method to Reduce Greenhouse Gas Emissions from Organic Waste in Mediterranean Climates.

[4] Vaish B., Srivastava V., Singh P., Singh A., Singh P.K., Singh R.P. (2016). Exploring untapped energy potential of urban solid waste. Energy Ecol. Environ..

[5] Heras-Saizarbitoria I., Zamanillo I., Laskurain I. (2013). Social acceptance of ocean wave energy: A case study of an OWC shoreline plant. Renew. Sustain. Energy Rev..

[6] Guo W.Q., Chen X.Y. (2011). Environmental challenge of risk society: A case study of Guangzhou residents’ opposition to the construction of waste incineration plant. Public Adm. Rev..

[7] Chen T., Yang Y. (2016). China’s Dilemma of Neighborhood Avoidance Effect—Investigating Residents Against Waste Incineration Power Generation Project. J. China Univ. Min. Technol..

[8] Hsu S.H. (2006). NIMBY opposition and solid waste incinerator siting in democratizing Taiwan. Soc. Sci. J..

[9] Liu Y., Ge Y.J., Xia B., Cui C.Y., Jiang X.Y., Martin S. (2019). Enhancing public acceptance towards waste-to-energy incineration projects: Lessons learned from a case study in China. Sustain. Cities Soc..

[10] Valinejadshoubi M., Ghanizadehgrayli M., Heidari S. (2018). Modeling and fabrication of a kinetic solar energy-absorbing window as a green idea for sustainable future buildings. J. Green Build..

[11] Wüstenhagen R., Wolsink M., Bürer M.J. (2007). Social acceptance of renewable energy innovation: An introduction to the concept. Energy Policy.

[12] Yuan X.L., Zuo J., Ma C.Y. (2011). Social acceptance of solar energy technologies in China—End users’ perspective. Energy Policy.

[13] Lim X.L., Lam W.H. (2014). Public acceptance of marine renewable energy in Malaysia. Energy Policy.

[14] Herman W.A.O., Herber R., Scholtens B. (2014). Not Under Our Back Yards? A case study of social acceptance of the Northern Netherlands CCS initiative. Renew. Sustain. Energy Rev..

[15] Yuan X.L., Zuo J., Ma R.J., Wang Y.T. (2015). How would social acceptance affect nuclear power development?. A Study from China. J. Clean. Prod..

[16] Chaliki P., Psomopoulos C.S., Themelis N.J. (2016). WTE plants installed in European cities: A review of success stories. Manag. Environ. Qual..

[17] Maqhuzu A.B., Yoshikawa K., Takahashi F. (2019). The effect of coal alternative fuel from municipal solid wastes employing hydrothermal carbonization on atmospheric pollutant emissions in Zimbabwe. Sci. Total Environ..

[18] Abdul Samad N.A.F., Jamin N.A., Saleh S. (2017). Torrefaction of Municipal Solid Waste in Malaysia. Energy Procedia.

[19] Dalmo F.C., Simão N.M., Lima H.Q.D., Medina Jimenez A.C., Nebra S., Martins G., Palacios-Bereche R., Henrique de Mello Sant’Ana P. (2019). Energy recovery overview of municipal solid waste in São Paulo State, Brazil. J. Clean. Prod..

[20] Bhuvaneswari V., Sudha G. (2015). A study on residents’ attitude towards household waste management with special reference to Coimbatore city. Int. Multidiscip. Res. J..

[21] Kang S., Cha J.H., Hong Y.J., Lee D., Kim K.H., Jeon E.C. (2017). Estimation of optimal biomass fraction measuring cycle for, municipal solid waste incineration facilities in Korea. Waste Manag..

[22] Rahardyan B., Matsuto T., Kakuta Y., Tanaka N. (2004). Resident’s concerns and attitudes towards solid waste management facilities. Waste Manag..

[23] Udawatta N., Zuo J., Chiveralls K., Yuan H.P., Zillante G., Elmualim A. (2018). Major factors impeding the implementation of waste management in Australian construction projects. J. Green Build..

[24] Lv J., Zhai X.Y. (2015). An International Comparison Study and Enlightenment of Waste Recycling System Based on Lateral Perspective. Ecol. Econ..

[25] Han Z.Y., Zeng D., Lie Q.B., Cheng C., Shi G.Z., Mou Z.S. (2019). Public willingness to pay and participate in domestic waste management in rural areas of China. Resour. Conserv. Recycl..

[26] Liu X.G., Wang Z.H., Li W., Li G.M., Zhang Y.Y. (2019). Mechanisms of public education influencing waste classification willingness of urban residents. Resour. Conserv. Recycl..

[27] Jones N., Evangelinos K., Halvadakis C.P., Iosifides I., Sophoulis C.M. (2010). Social factors influencing perceptions and willingness to pay for a market-based policy aiming on solid waste management. Resour. Conserv. Recycl..

[28] Lima M.L., Marques S. (2005). Towards successful social impact assessment follow-up: A case study of psychosocial monitoring of a solid waste incinerator in the North of Portugal. Impact Assess. Proj. Apprais..

[29] Heidari R., Yazdanparast R., Jabbarzadeh A. (2019). Sustainable design of a municipal solid waste management system considering waste separators: A real-world application. Sustain. Cities Soc..

[30] Lima M.L. (2006). Predictors of attitudes towards the construction of a waste incinerator: Two case studies. J. Appl. Soc. Psychol..

[31] Achillas C.H., Vlachokostas C.H., Moussiopoulos N., Banias G., Kafetzopoulos G., Karagiannidis A. (2011). Social acceptance for the development of a waste-to-energy plant in an urban area. Resour. Conserv. Recycl..

[32] Ryba J. (2014). The Influence of Planned Project KIC Odpady (WtE) in Karviná City on Residents’ Attitude and the Strength of NIMBY Effect. Master’s Theses.

[33] Sun C.W., Meng X.C., Peng S.J. (2017). Effects of Waste-to-Energy Plants on China’s Urbanization: Evidence from a Hedonic Price Analysis in Shenzhen. Sustainability.

[34] Navarro F., Carolina D.A., Marco R., Vincenzo T., Giovanni D.F. (2017). Social Surveys about Solid Waste Management within Higher Education Institutes: A Comparison. Sustainability.

[35] Liang J., Liu M. (2018). Network Design for Municipal Solid Waste Collection: A Case Study of the Nanjing Jiangbei New Area. Int. J. Environ. Res. Public Health.

[36] Zhang X.H. (2010). NIMBY of Waste Treatment Plant and Its Solution Mechanism of Social Conflict.

[37] Luo X.Z. (2011). The Analysis of NIMBY Way in Democratic Consultation Governance.

[38] Huang Y.L., Ning Y., Zhang T., Fei Y. (2015). Public acceptance of waste incineration power plants in China: Comparative case studies. Habitat Int..

[39] Bhuvaneshwari S., Hettiarachchi H., Meegoda N.J. (2019). Crop Residue Burning in India: Policy Challenges and Potential Solutions. Int. J. Environ. Res. Public Health.

[40] Ren X.Y., Che Y., Yang K., Tao Y. (2016). Risk perception and public acceptance toward a highly protested Waste-to-Energy facility. Waste Manag..

[41] Almasi A., Mohammadi M., Azizi A., Berizi Z., Shamsi K., Shahbazi A., Mosavi S.A. (2019). Assessing the knowledge, attitude and practice of the kermanshahi women towards reducing, recycling and reusing of municipal solid waste. Resour. Conserv. Recycl..

[42] Li Y.W., Vincent H., Martin D.J., Joop K. (2016). Government responses to environmental conflicts in urban China: The case of the Panyu waste incineration power plant in Guangzhou. J. Clean. Prod..

[43] Xu Y., Chan A.P.C., Xia B., Qian Q.K., Liu Y. (2015). Critical risk factors affecting the implementation of PPP waste-to-energy projects in China. Appl. Energy.

[44] Chu Z.J., Wang W.N., Wang B.R., Zhuang J. (2016). Research on Factors Influencing Municipal Household Solid Waste Separate Collection: Bayesian Belief Networks. Sustainability.

[45] The Ministry of Environmental Protection of China (2018). 2017 Annual report of solid waste pollution prevention and control in China. Renew. Resour. Recycl. Econ..

[46] Gui S., Zhao L.G., Zhang Z.J. (2019). Does municipal solid waste generation in China support the Environmental Kuznets Curve? New evidence from spatial linkage analysis. Waste Manag..

[47] (2016). The 13th Five-Year Construction Plan for National Urban Domestic Solid Waste Harmless Disposal Facilities. http://www.sdpc.gov.cn/zcfb/zcfbtz/201701/t20170122_836016.html.

[48] (2018). China Statistical Yearbook 2018.

[49] Bie R.S., Song X.F., Ji X.Y., Chen P., Liu Q.Q. (2013). Domestic and foreign domestic waste treatment status and policy. China Resour. Compr. Util..

[50] Tan M.Y. (2016). Comparative study on pollution control standards for domestic waste incineration at home and abroad. Sci. Technol. Innov..

[51] Heyer K.U., Stegmann R. (1987). Leachate management: Leachate generation, collection, treatment and costs. Phys. Rev. A.

[52] Viel J.F., Arveux P., Baverel J., Cahn J.Y. (2000). Soft-tissue sarcoma and non-Hodgkin’s lymphoma clusters around a municipal solid waste incinerator with high dioxin emission levels. Am. J. Epidemiol..

[53] Astrup T., Rosenblad C., Trapp S., Christensen T.H. (2005). Chromium release from waste incineration air-pollution-control residues. Environ. Sci. Technol..

[54] Ohnishi S., Fujii M., Ohata M., Rokuta I., Fujita T. (2018). Efficient energy recovery through a combination of waste-to-energy systems for a low-carbon city. Resour. Conserv. Recycl..

[55] Kang S., Kim S., Lee D., Lee J., Kim K.H., Jeon E.C. (2017). The Study on Biomass Fraction Estimation for Waste Incinerated in Korea: A Case Study. Sustainability.

